# The Association of Plasma Homocysteine Concentrations with a 10-Year Risk of All-Cause and Cardiovascular Mortality in a Community-Based Chinese Population

**DOI:** 10.3390/nu16121945

**Published:** 2024-06-19

**Authors:** Zhe Liang, Kaiyin Li, Hongyu Chen, Jia Jia, Jianping Li, Yong Huo, Fangfang Fan, Yan Zhang

**Affiliations:** 1 Department of Cardiology, Peking University First Hospital, No. 8 Xishiku Street, Xicheng District, Beijing 100034, China; liangzhechn@163.com (Z.L.); kaiyin0313@foxmail.com (K.L.); chy7449@163.com (H.C.); jiajia9985@163.com (J.J.); lijianping03455@pkufh.com (J.L.); huoyong@263.net.cn (Y.H.); 2 Institute of Cardiovascular Disease, Peking University First Hospital, No. 8 Xishiku Street, Xicheng District, Beijing 100034, China

**Keywords:** homocysteine, *MTHFR C677T* genetic polymorphism, mortality, cohort study, epidemiology

## Abstract

This study is aimed to examine the association of plasma homocysteine (Hcy) concentrations with a 10-year risk of all-cause and cardiovascular (CV) mortality and to explore the modification effect of methylenetetrahydrofolate reductase (*MTHFR*) *C677T* genetic polymorphism. This study included 5200 participants from a community-based Chinese population. Cox proportional hazard regression models were used to analyze the associations of Hcy and *MTHFR C677T* genotype with all-cause and CV mortality. The possible modification effect of the *MTHFR C677T* genotype on the Hcy–mortality relationship was assessed. The individuals with Hcy concentrations ≥ 10 μmol/L had a significantly higher risk of all-cause mortality compared to those with Hcy < 10 μmol/L (hazard ratio [HR]: 1.72, 95% confidence interval [CI]: 1.11–2.68, *p* = 0.015). The risk of CV mortality increased by 2% per 1 μmol/L Hcy increment (HR: 1.02, 95% CI: 1.00–1.03, *p* = 0.036). Despite the *MTHFR* genotype alone not being correlated with the mortality, the relationship between Hcy and all-cause mortality was significant in the *CC* genotype compared with *CT*/*TT* genotype (*p* for interaction = 0.036). Elevated plasma Hcy concentrations were associated with an increased 10-year risk of all-cause and CV mortality among the Chinese population. *MTHFR C677T* genetic polymorphism could modify the association between Hcy and all-cause mortality.

## 1. Introduction

Homocysteine (Hcy) is an intermediate thiol-containing nonproteinogenic amino acid derived from methionine catabolism, which is metabolized via remethylation or trans-sulfuration into cysteine [1]. Hcy plays a critical role in endothelial dysfunction, atherosclerosis, and thrombosis through various mechanisms, such as inflammation, lipid peroxidation, ferroptosis, and platelet activation [2,3,4]. Currently, the American Heart Association has recognized that elevated Hcy levels are associated with an increased risk of stroke and other vascular events [5].

Nevertheless, the evidence regarding the relationship between Hcy concentrations and the risk of death remains limited and conflicting, particularly in specific causes of death or populations. According to a meta-analysis of prospective studies, for each 5 μmol/L Hcy increment, the risk of all-cause and cardiovascular (CV) mortality increased by 27% and 32%, respectively [6]. Similarly, the risk of all-cause mortality increased by 33.6% with a 5 µmol/L increase in Hcy [7]. However, the causal relationships between Hcy and either all-cause or cause-specific mortality was not improved after the supplementation of folic acid or vitamin B in pooled Hcy-lowering clinical trials [8]. In specific populations, elevated Hcy was associated with the increased risk of all-cause mortality in participants with hypertension [9], diabetes [10], and central obesity [11], respectively, but the relationship between Hcy and all-cause mortality in patients with chronic kidney disease was not found [12].

Methylenetetrahydrofolate reductase (MTHFR) is a crucial regulatory enzyme within the one-carbon cycle. In individuals with the *C677T* variant (rs1801133), enzyme activity drops to around 67% and 25% for heterozygous (one copy) and homozygous (two copies) carriers of the T allele, respectively [13], which are associated with elevated plasma Hcy concentrations. Nevertheless, the *MTHFR 677TT* genotype did not have a significant effect on all-cause mortality [14,15], even in an inverse association with CV mortality [15]. In a Mendelian randomization analysis, the *MTHFR* polymorphism was utilized as an instrumental variable for estimating plasma Hcy concentrations [16]. However, the study did not provide evidence supporting a causal link between elevated plasma Hcy concentrations and all-cause or CV mortality.

Up to now, few prospective studies have considered the relationship between plasma Hcy concentrations and a risk of death in the Chinese general population. Multiple influencing factors, including genetic background, environmental exposures, lifestyles, and nutritional status, remain important knowledge gaps in this field. In particular, data are scarce regarding the *MTHFR C677T* genetic polymorphism alone and its inheritance–metabolism interaction concerning death endpoints in the general population. This study aims to examine the association of plasma Hcy concentrations with a 10-year risk of all-cause and CV mortality in a community-based longitudinal cohort in China. We further test the individual role as well as potential modification effect of *MTHFR C677T* genetic polymorphism, which may provide insight into the mechanisms underlying the relationship between Hcy and death outcomes.

## 2. Materials and Methods

### 2.1. Study Population

The subjects were recruited from an atherosclerosis cohort conducted in the Pingguoyuan and Gucheng communities in Beijing, China. The initial cohort investigated 9540 residents ≥ 40 years old from December 2011 to April 2012. The details of this study have been reported previously [17]. We further performed follow-up surveys until the end of 2021. Those with missing follow-up information, absent plasma Hcy concentrations, or lacking *MTHFR C677T* genotype were excluded, leaving 5200 participants in this final analysis. Ethical approval was obtained from the ethics committee of Peking University, and each participant signed an informed consent.

### 2.2. Demographic and Clinical Characteristics

Sociodemographic information, including age at the baseline, sex, lifestyle, and medical history, was collected through a standard questionnaire by trained medical professionals. Body mass index (BMI) was calculated as weight in kilograms divided by height in square meters. A detailed interview about tobacco use, which was defined as smoking at least one cigarette per day for more than half a year, and drinking alcohol, which was defined as drinking once or more per week for more than half a year, was also administered. Brachial blood pressure (BP) was obtained in an Omron HEM-7117 electronic sphygmomanometer after participants rested for 5 min, and the mean value of three consecutive measurements was taken for data analysis. Hypertension was defined as a self-reported history, a systolic BP (SBP) ≥ 140 mmHg or a diastolic BP (DBP) ≥ 90 mmHg, or the use of any antihypertensive drug. Diabetes was defined as a self-reported history, a fasting blood glucose concentration of the day ≥ 7 mmol/L, a 2 h oral glucose tolerance testing concentration ≥ 11.1 mmol/L, or the use of any hypoglycemic drug. Dyslipidemia was defined as a self-reported history, abnormal lipid profiles, or the use of any lipid-lowering drug. Cardiovascular disease (CVD) was defined as a self-reported history of myocardial infarction or stroke (including transient ischemic attack).

### 2.3. Specimen Collection and Laboratory Testing

Blood specimens were obtained via venipuncture after an overnight fast, which were separated into plasma and serum samples within 30 min of collection and then frozen at −80 °C. The Roche C8000 Automatic Biochemical Analyzer detected blood glucose, total cholesterol, triglycerides, low-density lipoprotein cholesterol, high-density lipoprotein cholesterol, and creatinine in serum samples. Estimated glomerular filtration rate (eGFR) was calculated derived from the consensus of Chronic Kidney Disease Epidemiology Collaboration (CKD-EPI) [18], and eGFR ≥ 90 mL/min/1.73m^2^ was considered as normal renal function.

Plasma Hcy was assayed at baseline by the enzymatic cycling method using the Beckman Coulter AU480 Automatic Biochemical Analyzer. Serum folate was measured by the electrochemiluminescence immunoassay in a commercial laboratory (New Industrial, Shenzhen, China). The standardized procedures for measuring Hcy and folate levels have been established in a previous study [19]. The *MTHFR C677T* genotype of all subjects could be obtained by retrieving Asian ExomeChip, just as a specially designed exome array based on the Infinium Human Exome BeadChip (Illumina, San Diego, CA, USA). The design details of Asian ExomeChip have been described previously [20,21].

### 2.4. Ascertainment of Mortality

Data on participants’ deaths were collected from the Chinese Center for Disease Control and Prevention (National Mortality Surveillance System) and Beijing Municipal Health Commission (Inpatient Medical Record Home Page System). The International Classification of Diseases in 10th Revision (ICD-10) was used to classify the leading cause of death. The primary endpoint was all-cause death. The secondary endpoint was CV death (I00-I99). We defined the follow-up time from baseline to the death of participants or the end of follow-up (31 December 2021).

### 2.5. Statistical Analysis

The baseline characteristics of the entire community cohort and stratified by clinical common thresholds of plasma Hcy concentrations, according to the different definitions of hyperhomocysteinemia (HHcy) [22,23], were summarized in mean (standard deviation, SD) or median (interquartile range, IQR) for quantitative variables and frequencies for categorical variables. Characteristic differences between the groups were tested in one-way analysis of variance (ANOVA) together with Kruskal–Wallis test for quantitative variables and χ^2^ analysis for categorical variables as appropriate. The Kaplan–Meier method was used to estimate the cumulative hazards of death stratified by Hcy concentrations and *MTHFR C677T* genotype, and group differences were calculated by log-rank tests. Cox proportional hazard regression models were used to analyze the associations of plasma Hcy concentrations and *MTHFR C677T* genotype with the risk of all-cause and CV mortality, which were adjusted for several potential confounding factors, including baseline age, sex, BMI, eGFR, smoking, drinking, hypertension, diabetes, dyslipidemia, CVD, taking antihypertensive drugs, hypoglycemic drugs, lipid-lowering drugs, and serum folate levels. Furthermore, the dose–response associations between Hcy and endpoints were examined with restricted cubic spline (RCS) in the fully adjusted models. Additionally, possible modifications to the relationships between plasma Hcy concentrations and outcomes were assessed for variables including *MTHFR C677T* genotype, serum folate levels, and some of the covariates mentioned above. A two-tailed *p*-value < 0.05 was considered statistical significance. All analyses were conducted in R (http://www.R-project.org, accessed on 12 March 2024).

## 3. Results

### 3.1. Baseline Characteristics

The characteristics of the overall cohort and the comparison of participants stratified by clinical thresholds of plasma Hcy concentrations (<10, ≥10 to <15, ≥15 μmol/L) are shown in Table 1. The mean (SD) age of the subjects was 57.14 (8.93) years old, and 62.1% (*n* = 3230) were female. The mean (SD) BMI and eGFR of the participants were 26.09 (3.38) kg/m^2^ and 94.28 (13.13) mL/min/1.73m^2^. The median (IQR) value of plasma Hcy was 11.95 (10.00, 14.89) μmol/L. The number of subjects with each *MTHFR C677T* genotype (*CC*, *CT*, and *TT*) accounted for 18.5% (*n* = 963), 46.7% (*n* = 2427), and 34.8% (*n* = 1810), respectively. The median (IQR) values in different groups of plasma Hcy were 8.82 (8.08, 9.49) μmol/L, 11.97 (11.02, 13.23) μmol/L, and 18.63 (16.51, 25.19) μmol/L. Participants with higher Hcy concentrations were more likely to be older, be males, have a higher BMI, have lower serum folate levels, have a higher proportion of the *MTHFR 677TT* genotype, use tobacco, drink, take antihypertensive drugs, and present with impaired renal function, hypertension, and CVD. No differences were observed in diabetes, dyslipidemia, and taking lipid-lowering drugs.

### 3.2. All-Cause and CV Mortality

**Figure 1:**
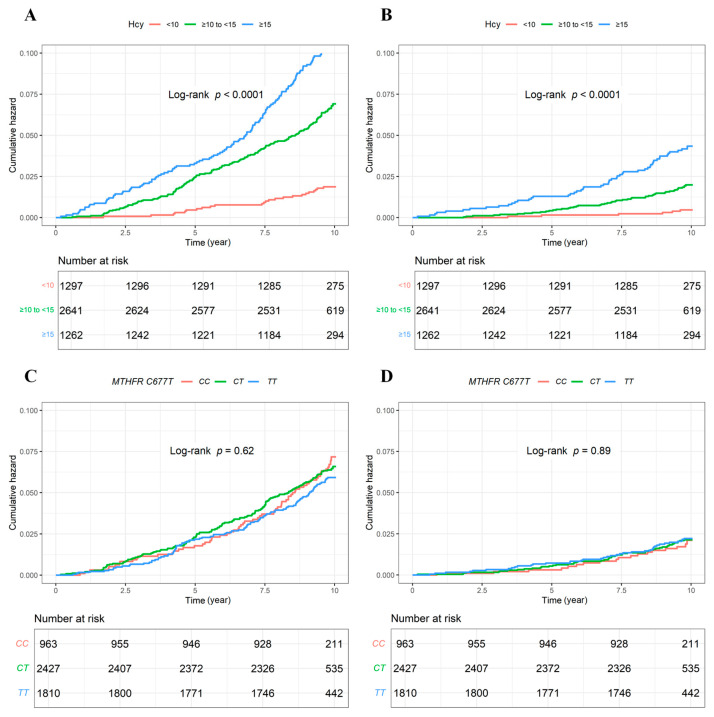
Kaplan–Meier survival curves for the cumulative hazards of mortality. (**A**): stratified by clinical thresholds of plasma Hcy concentrations in all-cause mortality; (**B**): stratified by clinical thresholds of plasma Hcy concentrations in CV mortality; (**C**): stratified by the *MTHFR C677T* genotype in all-cause mortality; (**D**): stratified by the *MTHFR C677T* genotype in CV mortality. CV: cardiovascular; Hcy: homocysteine; MTHFR: methylenetetrahydrofolate reductase.

During a mean (SD) follow-up of 9.65 (1.11) years, 320 deaths were documented, covering 107 CV deaths. Kaplan–Meier survival curves (Figure 1) revealed a significant dose–response relationship of Hcy with all-cause and CV mortality (all log-rank tests *p* < 0.001). In other words, the participants with Hcy concentrations < 10 μmol/L had the lowest risk of all-cause and CV mortality. Meanwhile, there was no significant difference for the cumulative hazards of all-cause and CV mortality stratified by the *MTHFR C677T* genotype (all log-rank tests *p* > 0.05).

The results of the Cox proportional hazard regression analyses are summarized in Table 2. After adjusting for covariates, using Hcy concentrations < 10 μmol/L as a reference, the group with Hcy concentrations ≥ 10 to < 15 μmol/L and the group with Hcy concentrations ≥ 15 μmol/L had an increased risk of all-cause mortality by 74% (hazard ratio [HR]: 1.74, 95% confidence interval [CI]: 1.12–2.71, *p* = 0.014) and 64% (HR: 1.64, 95% CI: 1.01–2.68, *p* = 0.047), respectively. We further merged aforesaid clinical thresholds: participants with Hcy concentrations ≥ 10 μmol/L also had a significantly higher risk of all-cause mortality compared to those with Hcy < 10 μmol/L (HR: 1.72, 95% CI: 1.11–2.68, *p* = 0.015). The risk of CV mortality increased by 2% per 1 μmol/L increment of plasma Hcy concentrations (HR: 1.02, 95% CI: 1.00–1.03, *p* = 0.036), and a similar increasing trend was observed across the three groups by clinical thresholds of Hcy. Differently, the association of the *MTHFR C677T* genotype with the 10-year risk of all-cause and CV mortality was not statistically significant.

**Figure 2:**
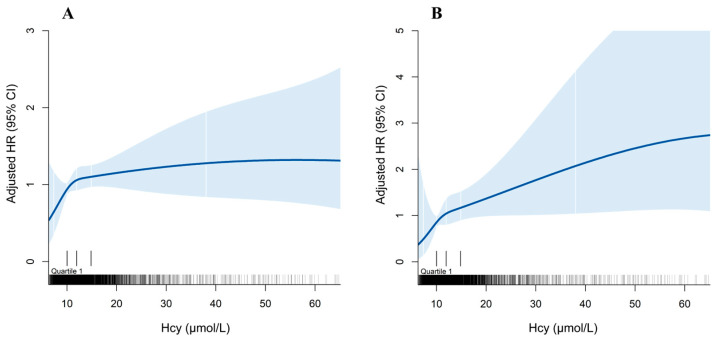
The restricted cubic spline of the dose–response relationship between plasma Hcy concentrations and mortality. (**A**): plasma Hcy concentrations and all-cause mortality; (**B**): plasma Hcy concentrations and CV mortality. Restricted cubic spline was adjusted for baseline age, sex, BMI, eGFR, smoking, drinking, hypertension, diabetes, dyslipidemia, CVD, taking antihypertensive drugs, hypoglycemic drugs, lipid-lowering drugs, and serum folate levels. The blue area indicates the 95% CI for the restricted cubic spline. The X-axis is truncated at the 0.5th and 99.5th percentiles of plasma Hcy concentrations. BMI: body mass index; CI: confidence interval; CV: cardiovascular; CVD: cardiovascular disease; eGFR: estimated glomerular filtration rate; Hcy: homocysteine; HR: hazard ratio.

We further conducted RCS using fully adjusted models to explicitly show the dose–response associations between Hcy and different outcomes (Figure 2). The smooth curves indicated that there were rising trends for the risk of both all-cause and CV mortality with increased plasma Hcy concentrations.

### 3.3. Stratification Analyses

We performed stratified analyses based on the clinical threshold of 10 μmol/L for plasma Hcy concentrations. A significant modifying effect of *MTHFR C677T* genetic polymorphism on the association between Hcy and all-cause mortality was observed (*p* for interaction = 0.036) (Table 3). Compared with the *CT*/*TT* genotype, elevated Hcy concentrations were significantly associated with an increased risk of all-cause mortality in individuals with the *MTHFR 677CC* genotype (HR: 5.24, 95% CI: 1.27–21.63). Additionally, the positive association between plasma Hcy concentrations and all-cause mortality appeared to be stronger in the groups of age below 65 years old, those with a BMI < 24 kg/m^2^, those with an eGFR ≥ 90 mL/min/1.73m^2^, non-smokers, non-drinkers, non-diabetics, those not taking antihypertensive drugs, those not taking hypoglycemic drugs, those not taking lipid-lowering drugs, and those with serum folate concentrations ≥ 6.18 ng/mL, but the interaction test for a trend was not significant (all *p* for interaction > 0.05).

In terms of CV death, among the existing subgroup variables, we did not find any potential effect modifier for the relationship between Hcy concentrations and CV mortality (all *p* for interaction > 0.05) (Table S1).

## 4. Discussion

In this large community-based longitudinal study with 5200 Chinese residents, we found that higher plasma Hcy concentrations but not the *MTHFR C677T* genotype were associated with an increased 10-year risk of all-cause and CV mortality. However, it is noteworthy that *MTHFR C677T* genetic polymorphism played a vital role in the relationship between Hcy and the 10-year risk of all-cause mortality.

After adjusting for multiple covariates, Hcy was positively associated with all-cause mortality. It was worth mentioning that we further adjusted for serum folate levels based on previous studies, which have highlighted the significant role of folate in the degradation of Hcy and have also demonstrated its correlation with all-cause and CV mortality [24,25]. Some previous studies showed consistent results that plasma Hcy was associated with all-cause mortality in Western populations or participants with a history of CVD, respectively [26,27]. Another prospective cohort study in China also revealed that elevated Hcy levels were associated with a higher risk of all-cause mortality during a median follow-up of 5.69 years [28]. Unlike our study, this study focused on retired workers (average age over 60 years), resulting in more adverse events that occurred during a relatively short follow-up period. Thus, our data further substantiate the correlation between Hcy and all-cause mortality in a broader population category.

The definition of HHcy has been a subject of debate. It is sometimes defined as plasma Hcy concentrations ≥ 15 μmol/L [22]; however, the American Heart Association and American Stroke Association utilized a lower cut-off (≥10 μmol/L) to classify HHcy [23]. Compared to essential hypertension alone, hypertension accompanied by plasma Hcy concentrations ≥ 10 μmol/L (defined as H-type hypertension) has the potential to exacerbate significantly the vascular damage induced by hypertension, consequently raising the risk of CVD complications and all-cause mortality [29]. In this study, we observed that compared to a reference value of Hcy concentrations < 10 μmol/L, whether considering separate clinical thresholds or pooled clinical thresholds, Hcy ≥ 10 μmol/L consistently exhibited a higher risk of all-cause mortality. Therefore, consistent with the previously established lower threshold, we believe that plasma Hcy concentrations ≥ 10 µmol/L should be taken seriously and that early intervention is necessary to reduce the subsequent risk of mortality.

Regarding cause-specific mortality, we found a positive and linear relationship between plasma Hcy and CV mortality. Although the critical risk factor of Hcy that predisposes patients to the process of CVD may be distinct from those that contribute to CV death, people who experience CV death are the most possible to have CVD listed as a potential cause of death. In brief, multiple molecular mechanisms underlying Hcy may contribute to the occurrence and development of CVD, including damaging vascular endothelial cells, promoting vascular smooth muscle cell proliferation and migration, and activating excessive oxidative stress reactions, lipid metabolism disorders, and coagulation dysfunction [29]. Numerous studies have demonstrated that increased plasma Hcy may bring about cardio- and cerebrovascular diseases, neurological disorders, and even target organ damage [30,31,32,33]. This association between elevated Hcy levels and higher CV mortality was similar in both Korean [24] and Japanese [34] general populations, which confirms that the association between them is robust and consistent in East Asian populations.

The *MTHFR* polymorphism involves the mutation of cytosine to thymine at position 677. This would increase Hcy accumulation, reduce methylation capacity, alternate inflammatory mediators, and damage vascular endothelium [35]. As a consequence, long-term harm may increase CV death synergistically along with multiple adverse mechanisms. However, our results did not support the association between *MTHFR C677T* genetic polymorphism and 10-year all-cause and CV mortality. In agreement with our results, the *MTHFR 677TT* genotype was not correlated with all-cause mortality either in four individual cohorts or in the pooled study population without mandatory folic acid fortification in Denmark [14]. One possible explanation is that the complex interactions of multiple genetic and environmental factors exert more significant influence on disease susceptibility and death than the purely unfavorable variant of *C677T* polymorphism.

Despite the *MTHFR* genotype alone not being correlated with the mortality mentioned above, insight into what degree of the *MTHFR* genotype could modify the Hcy–mortality relationship yields novelty. In this study, the positive association between plasma Hcy concentrations and all-cause mortality was observed only in the *MTHFR 677CC* genotype compared with the *CT*/*TT* genotype. The effect modification of the *MTHFR C677T* genotype for the relationship between Hcy concentrations and CV mortality showed a similar trend but no interaction, possibly due to fewer adverse events occurring. In another post hoc analysis of the China Stroke Primary Prevention Trial, the positive relationship between Hcy and all-cause mortality was significant in the *CC*/*CT* genotype but not in the *TT* genotype among Chinese hypertensive patients [36], which provides comparable clinical implications to researchers. In our participants with the *CC* genotype, the standardized mean differences (SMDs) between those with HHcy and those with normal Hcy concentrations were higher than that in the *CT*/*TT* genotype across multiple features (Table S2). This suggests that elevated Hcy concentrations in individuals with the *CC* genotype may indicate a poorer overall health status, potentially contributing to increased all-cause mortality. This finding provides a theoretical basis for the precise treatment and prevention of HHcy with different *MTHFR C677T* genotypes.

To our knowledge, this is the first large-scale cohort study to examine the *MTHFR C677T* genetic polymorphism–Hcy metabolism interaction in death endpoints through long-term follow-up among the general population, which may offer a vital clue of gene-environment relevance and guide future research. This present study has some limitations. First, as this study is a single-center study based on a northern Chinese population, our findings need to be validated by measuring plasma Hcy concentrations and the *MTHFR C677T* genotype in other independent populations. Second, plasma Hcy concentrations were assayed at the baseline timepoint and may not accurately reflect long-term changes in Hcy. Many studies faced a similar limitation, and the impact of changes in Hcy over time requires further investigation. Third, the COVID-19 pandemic overlapped with our research period from 2020 to 2021 and may have an effect on the all-cause mortality. However, its impact could be considered negligible, as China experienced extremely low life expectancy losses, COVID-19 mortality, and excess mortality [37,38]. Lastly, we focused on a limited range of mortality types. The individual effects of Hcy and the *MTHFR C677T* genetic polymorphism, as well as its modifying role, on non-CV mortality, such as cancer, remain poorly understood and require in-depth research in the future.

## 5. Conclusions

In conclusion, our study provided solid evidence to support the positive relationship of plasma Hcy concentrations with 10-year all-cause and CV mortality in a community-based Chinese population. We also found that elevated Hcy was associated with the increased all-cause mortality in people with the *MTHFR 677CC* genotype. In contrast, the relationship faded away in those with the *CT*/*TT* genotype. This study provides novel insights into Hcy–*MTHFR* interaction on mortality in the Chinese population. Revealing the underlying biological mechanisms and observing the impact of Hcy-lowering therapy on long-term mortality warrant further investigation.

## Figures and Tables

**Table 1:** Characteristics of the participants at baseline.

Characteristics	Overall	Plasma Hcy Concentrations			*p*-Value
		<10 μmol/L	≥10 to <15 μmol/L	≥15 μmol/L	
N	5200	1297	2641	1262	
Age (year), mean (SD)	57.14 (8.93)	53.79 (7.47)	57.81 (8.67)	59.18 (9.88)	<0.001
Sex, N (%)					<0.001
Male	1970 (37.9)	137 (10.6)	962 (36.4)	871 (69.0)	
Female	3230 (62.1)	1160 (89.4)	1679 (63.6)	391 (31.0)	
Plasma Hcy (μmol/L), median (IQR)	11.95 (10.00, 14.89)	8.82 (8.08, 9.49)	11.97 (11.02, 13.23)	18.63 (16.51, 25.19)	<0.001
Serum folate (ng/mL), median (IQR)	6.18 (4.98, 8.19)	7.70 (6.05, 10.23)	6.32 (5.20, 8.07)	4.88 (4.22, 5.89)	<0.001
*MTHFR C677T*, N (%)					<0.001
*CC*	963 (18.5)	322 (24.8)	503 (19.0)	138 (10.9)	
*CT*	2427 (46.7)	686 (52.9)	1325 (50.2)	416 (33.0)	
*TT*	1810 (34.8)	289 (22.3)	813 (30.8)	708 (56.1)	
BMI (kg/m^2^), mean (SD)	26.09 (3.38)	25.95 (3.52)	26.08 (3.34)	26.28 (3.28)	0.046
eGFR (mL/min/1.73 m^2^), mean (SD)	94.28 (13.13)	101.18 (9.15)	94.03 (11.63)	87.73 (15.79)	<0.001
eGFR classification (mL/min/1.73 m^2^), N (%)					<0.001
≥90	3606 (69.4)	1147 (88.6)	1816 (68.8)	643 (51.0)	
<90	1590 (30.6)	148 (11.4)	824 (31.2)	618 (49.0)	
Current smoking, N (%)	1025 (19.7)	82 (6.3)	470 (17.8)	473 (37.5)	<0.001
Current drinking, N (%)	1230 (23.7)	143 (11.0)	581 (22.0)	506 (40.1)	<0.001
Prevalence of disease, N (%)					
Hypertension	2657 (51.1)	547 (42.2)	1362 (51.6)	748 (59.3)	<0.001
Diabetes	1289 (24.8)	304 (23.4)	687 (26.0)	298 (23.6)	0.115
Dyslipidemia	3733 (71.8)	930 (71.7)	1911 (72.4)	892 (70.7)	0.551
CVD	292 (5.6)	49 (3.8)	143 (5.4)	100 (7.9)	<0.001
Medication, N (%)					
Antihypertensive drugs	1689 (32.7)	346 (26.8)	865 (33.0)	478 (38.1)	<0.001
Hypoglycemic drugs	572 (11.0)	141 (10.9)	321 (12.2)	110 (8.8)	0.006
Lipid-lowering drugs	555 (10.8)	141 (11.0)	288 (11.0)	126 (10.1)	0.643
Death endpoint, N (%)					
All-cause mortality	320 (6.2)	24 (1.9)	172 (6.5)	124 (9.8)	<0.001
CV mortality	107 (2.1)	6 (0.5)	50 (1.9)	51 (4.0)	<0.001

BMI: body mass index; CV: cardiovascular; CVD: cardiovascular disease; eGFR: estimated glomerular filtration rate; Hcy: homocysteine; IQR: interquartile range; MTHFR: methylenetetrahydrofolate reductase; SD: standard deviation.

**Table 2:** Associations of plasma Hcy concentrations and *MTHFR C677T* genotype with all-cause and CV mortality.

Endpoints	N	No. of Deaths, N (%)	Crude		Adjusted *	
			HR (95% CI)	*p*-Value	HR (95% CI)	*p*-Value
All-cause mortality						
Linear trend						
Hcy	5200	320 (6.2)	1.02 (1.01, 1.03)	<0.001	1.01 (0.99, 1.02)	0.363
Classified clinical threshold						
<10 μmol/L	1297	24 (1.9)	ref		ref	
≥10 to <15 μmol/L	2641	172 (6.5)	3.61 (2.35, 5.53)	<0.001	1.74 (1.12, 2.71)	0.014
≥15 μmol/L	1262	124 (9.8)	5.55 (3.58, 8.59)	<0.001	1.64 (1.01, 2.68)	0.047
Pooled clinical threshold						
<10 μmol/L	1297	24 (1.9)	ref		ref	
≥10 μmol/L	3903	296 (7.6)	4.23 (2.79, 6.41)	<0.001	1.72 (1.11, 2.68)	0.015
*MTHFR C677T*						
*CC*	963	64 (6.6)	ref		ref	
*CT*	2427	152 (6.3)	0.94 (0.71, 1.27)	0.702	1.05 (0.78, 1.42)	0.729
*TT*	1810	104 (5.7)	0.86 (0.63, 1.18)	0.350	1.03 (0.75, 1.42)	0.850
CV mortality						
Linear trend						
Hcy	5200	107 (2.1)	1.03 (1.01, 1.04)	<0.001	1.02 (1.00, 1.03)	0.036
Classified clinical threshold						
<10 μmol/L	1297	6 (0.5)	ref		ref	
≥10 to <15 μmol/L	2641	50 (1.9)	4.20 (1.80, 9.81)	<0.001	1.80 (0.76, 4.30)	0.183
≥15 μmol/L	1262	51 (4.0)	9.16 (3.93, 21.34)	<0.001	2.06 (0.81, 5.20)	0.128
Pooled clinical threshold						
<10 μmol/L	1297	6 (0.5)	ref		ref	
≥10 μmol/L	3903	101 (2.6)	5.78 (2.54, 13.18)	<0.001	1.85 (0.78, 4.39)	0.160
*MTHFR C677T*						
*CC*	963	18 (1.9)	ref		ref	
*CT*	2427	50 (2.1)	1.10 (0.64, 1.89)	0.717	1.34 (0.77, 2.31)	0.297
*TT*	1810	39 (2.2)	1.15 (0.66, 2.01)	0.626	1.47 (0.83, 2.61)	0.186

* Cox proportional hazard regression models were adjusted for baseline age, sex, BMI, eGFR, smoking, drinking, hypertension, diabetes, dyslipidemia, CVD, taking antihypertensive drugs, hypoglycemic drugs, lipid-lowering drugs, and serum folate levels. BMI: body mass index; CI: confidence interval; CV: cardiovascular; CVD: cardiovascular disease; eGFR: estimated glomerular filtration rate; Hcy: homocysteine; HR: hazard ratio; MTHFR: methylenetetrahydrofolate reductase.

**Table 3:** The stratified analysis of the association between plasma Hcy concentrations and all-cause mortality.

Subgroup	N	No. of Deaths, N (%)		HR (95% CI) *	*p* for Interaction
		Hcy < 10 μmol/L	Hcy ≥ 10 μmol/L		
Age (year)					0.530
<65	4158	12 (1.0)	103 (3.5)	1.93 (1.04, 3.56)	
≥65	1042	12 (10.2)	193 (20.9)	1.47 (0.81, 2.69)	
Sex					0.805
Male	1970	5 (3.6)	189 (10.3)	1.86 (0.76, 4.58)	
Female	3230	19 (1.6)	107 (5.2)	1.64 (0.99, 2.71)	
BMI (kg/m^2^)					0.496
<24	1402	6 (1.6)	100 (9.8)	2.48 (1.07, 5.73)	
≥24 to <28	2407	12 (2.0)	133 (7.3)	1.39 (0.75, 2.57)	
≥28	1391	6 (1.9)	63 (5.9)	1.48 (0.63, 3.46)	
eGFR (mL/min/1.73 m^2^)					0.608
≥90	3606	15 (1.3)	102 (4.1)	1.83 (1.05, 3.20)	
<90	1590	9 (6.1)	194 (13.5)	1.46 (0.73, 2.90)	
Current smoking					0.262
No	4175	23 (1.9)	204 (6.9)	1.57 (1.00, 2.47)	
Yes	1025	1 (1.2)	92 (9.8)	4.24 (0.59, 30.53)	
Current drinking					0.768
No	3970	21 (1.8)	208 (7.4)	1.73 (1.08, 2.77)	
Yes	1230	3 (2.1)	88 (8.1)	1.43 (0.45, 4.57)	
Hypertension					0.772
No	2543	8 (1.1)	79 (4.4)	1.84 (0.88, 3.88)	
Yes	2657	16 (2.9)	217 (10.3)	1.62 (0.95, 2.74)	
Diabetes					0.496
No	3911	12 (1.2)	174 (6.0)	1.94 (1.06, 3.55)	
Yes	1289	12 (3.9)	122 (12.4)	1.45 (0.78, 2.67)	
Dyslipidemia					0.543
No	1467	5 (1.4)	88 (8.0)	2.14 (0.86, 5.35)	
Yes	3733	19 (2.0)	208 (7.4)	1.57 (0.96, 2.57)	
Antihypertensive drugs					0.370
No	3481	11 (1.2)	149 (5.9)	2.05 (1.09, 3.86)	
Yes	1689	13 (3.8)	145 (10.8)	1.40 (0.78, 2.51)	
Hypoglycemic drugs					0.753
No	4614	17 (1.5)	233 (6.7)	1.76 (1.05, 2.96)	
Yes	572	7 (5.0)	63 (14.6)	1.52 (0.69, 3.36)	
Lipid-lowering drugs					0.131
No	4597	18 (1.6)	259 (7.5)	1.96 (1.19, 3.24)	
Yes	555	6 (4.3)	34 (8.2)	0.88 (0.36, 2.11)	
*MTHFR C677T*					0.036
*CC*	963	2 (0.6)	62 (9.7)	5.24 (1.27, 21.63)	
*CT*/*TT*	4237	22 (2.3)	234 (7.2)	1.38 (0.87, 2.19)	
Serum folate (ng/mL)					0.104
<6.18	2599	10 (2.8)	174 (7.7)	1.08 (0.56, 2.08)	
≥6.18	2601	14 (1.5)	122 (7.4)	2.22 (1.26, 3.91)	

* Cox proportional hazard regression models were adjusted for baseline age, sex, BMI, eGFR, smoking, drinking, hypertension, diabetes, dyslipidemia, CVD, taking antihypertensive drugs, hypoglycemic drugs, lipid-lowering drugs, and serum folate levels. When a variable was assessed for its possible modification on the relationship of plasma Hcy concentrations and all-cause mortality, the stratified variable was not adjusted repeatedly. The stratified analysis for CVD is not feasible due to the limited number of death events in those with Hcy < 10 μmol/L. BMI: body mass index; CI: confidence interval; CVD: cardiovascular disease; eGFR: estimated glomerular filtration rate; Hcy: homocysteine; HR: hazard ratio; MTHFR: methylenetetrahydrofolate reductase.

## Data Availability

The datasets analyzed or generated during the study are available from the corresponding authors on reasonable request.

## Supplementary Materials

The following supporting information can be downloaded at https://www.mdpi.com/article/10.3390/nu16121945/s1, Table S1: Stratified analysis of the association between plasma Hcy concentrations and CV mortality; Table S2: The baseline characteristics of the participants grouped by plasma Hcy concentrations and the *MTHFR C677T* genotype.
