# Supplementary material for: The Association of Plasma Homocysteine Concentrations with a 10-Year Risk of All-Cause and Cardiovascular Mortality in a Community-Based Chinese Population
Source: Nutrients. 2024 Jun 19;16(12):1945. doi: 10.3390/nu16121945 (PMC11206274; doi:10.3390/nu16121945)
Supplement: Supplementary file 1 [file nutrients-16-01945-s001.zip › nutrients-3006171-supplementary.pdf]

The Association of Plasma Homocysteine Concentrations with a 10-Year Risk of All-Cause and Cardiovascular Mortality in a Community-Based Chinese Population

Supplementary Materials

Table S1 Stratified analysis of the association between plasma Hcy concentrations and CV mortality

Subgroup	N	No. of deaths, N (%)		HR (95% CI)*	*P* for interaction
		Hcy < 10 μmol/L	Hcy ≥ 10 μmol/L		
Age (year)					0.711
< 65	4158	2 (0.2)	24 (0.8)	2.36 (0.55, 10.18)	
≥ 65	1042	4 (3.4)	77 (8.3)	1.70 (0.60, 4.81)	
Sex					0.617
Male	1970	1 (0.7)	68 (3.7)	2.89 (0.39, 21.22)	
Female	3230	5 (0.4)	33 (1.6)	1.70 (0.65, 4.43)	
BMI (kg/m^2)					0.223
< 24	1402	1 (0.3)	36 (3.5)	4.48 (0.60, 33.34)	
≥ 24 to < 28	2407	2 (0.3)	47 (2.6)	2.40 (0.57, 10.19)	
≥ 28	1391	3 (0.9)	18 (1.7)	0.71 (0.20, 2.49)	
eGFR ($mL/min/1.73m^2$)					0.840
≥ 90	3606	3 (0.3)	24 (1.0)	2.04 (0.60, 6.88)	
< 90	1590	3 (2.0)	77 (5.3)	1.72 (0.52, 5.63)	
Current drinking					0.646
No	3970	5 (0.4)	71 (2.5)	2.06 (0.81, 5.28)	
Yes	1230	1 (0.7)	30 (2.8)	1.20 (0.16, 8.94)	
Diabetes					0.781
No	3911	3 (0.3)	49 (1.7)	1.69 (0.51, 5.59)	
Yes	1289	3 (1.0)	52 (5.3)	2.13 (0.65, 7.04)	
Dyslipidemia					0.793
No	1467	1 (0.3)	22 (2.0)	2.41 (0.32, 18.27)	
Yes	3733	5 (0.5)	79 (2.8)	1.81 (0.71, 4.64)	
Antihypertensive drugs					0.212
No	3481	1 (0.1)	39 (1.5)	4.72 (0.64, 35.08)	
Yes	1689	5 (1.4)	61 (4.5)	1.36 (0.53, 3.50)	
Lipid-lowering drugs					0.341
No	4597	4 (0.4)	86 (2.5)	2.39 (0.85, 6.74)	
Yes	555	2 (1.4)	14 (3.4)	0.95 (0.21, 4.30)	
MTHFR C677T					0.836
CC	963	1 (0.3)	17 (2.7)	2.26 (0.29, 17.34)	
CT/TT	4237	5 (0.5)	84 (2.6)	1.80 (0.70, 4.60)	
Serum folate (ng/mL)					0.070
< 6.18	2599	4 (1.1)	62 (2.8)	0.84 (0.29, 2.40)	
≥ 6.18	2601	2 (0.2)	39 (2.4)	4.01 (0.95, 16.85)	

*Cox proportional hazard regression models were adjusted for baseline age, sex, BMI, eGFR, smoking, drinking, hypertension, diabetes, dyslipidemia, CVD, taking antihypertensive drugs, hypoglycemic drugs, lipid-lowering drugs and serum folate levels. When a variable was assessed

for its possible modification on the relationship of plasma Hcy concentrations and CV mortality, the stratified variable was not adjusted repeatedly. The stratified analyses for smoking, hypertension, CVD and taking hypoglycemic drugs are not feasible due to the limited number of death events in those with Hcy < 10 μmol/L. BMI: body mass index; CI: confidence interval; CV: cardiovascular; CVD: cardiovascular disease; eGFR: estimated glomerular filtration rate; Hcy: homocysteine; HR: hazard ratio; MTHFR: methylenetetrahydrofolate reductase.

Table S2 The baseline characteristics of the participants grouped by plasma Hcy concentrations and the *MTHFR C677T* genotype

Characteristics	Overall	***CC***		SMD	*P* value	***CT/TT***		SMD	*P* value
		Hcy < 10 μmol/L	Hcy ≥ 10 μmol/L			Hcy < 10 μmol/L	Hcy ≥ 10 μmol/L		
N	5200	322	641			975	3262		
Age (year), mean (SD)	57.14 (8.93)	53.63 (7.49)	59.73 (9.12)	0.73 (0.59, 0.87)	<0.001	53.85 (7.47)	57.97 (9.07)	0.50 (0.42, 0.57)	<0.001
Sex, N (%)				0.92 (0.78, 1.05)	<0.001			0.88 (0.81, 0.95)	<0.001
Male	1970 (37.9)	40 (12.4)	328 (51.2)			97 (9.9)	1505 (46.1)		
Female	3230 (62.1)	282 (87.6)	313 (48.8)			878 (90.1)	1757 (53.9)		
Plasma Hcy (μmol/L), median (IQR)	11.95 (10.00, 14.89)	8.75 (8.04, 9.45)	12.58 (11.17, 14.66)	1.36 (1.22, 1.51)	<0.001	8.85 (8.10, 9.49)	13.29 (11.52, 16.79)	1.09 (1.02, 1.17)	<0.001
Serum folate (ng/mL), median (IQR)	6.18 (4.98, 8.19)	8.28 (6.31, 10.33)	6.23 (5.22, 8.31)	0.34 (0.21, 0.48)	<0.001	7.44 (5.96, 10.15)	5.65 (4.69, 7.37)	0.16 (0.09, 0.24)	<0.001
BMI (kg/m^2), mean (SD)	26.09 (3.38)	25.95 (3.51)	25.88 (3.14)	0.02 (-0.12, 0.15)	0.782	25.95 (3.53)	26.19 (3.36)	0.07 (-0.00, 0.14)	0.053
eGFR (mL/min/1.73m^2), mean (SD)	94.28 (13.13)	100.72 (9.07)	89.79 (14.39)	0.91 (0.77, 1.05)	<0.001	101.33 (9.18)	92.42 (13.21)	0.78 (0.71, 0.86)	<0.001
eGFR classification (mL/min/1.73m^2), N (%)				0.70 (0.56, 0.84)	<0.001			0.62 (0.54, 0.69)	<0.001
≥ 90	3606 (69.4)	280 (87.0)	365 (57.1)			867 (89.1)	2094 (64.2)		
< 90	1590 (30.6)	42 (13.0)	274 (42.9)			106 (10.9)	1168 (35.8)		
Current smoking, N (%)	1025 (19.7)	22 (6.8)	155 (24.2)	0.49 (0.36, 0.63)	<0.001	60 (6.2)	788 (24.2)	0.52 (0.45, 0.59)	<0.001
Current drinking, N (%)	1230 (23.7)	42 (13.0)	166 (25.9)	0.33 (0.19, 0.46)	<0.001	101 (10.4)	921 (28.2)	0.47 (0.39, 0.54)	<0.001
Prevalence of disease, N (%)									
Hypertension	2657 (51.1)	129 (40.1)	370 (57.7)	0.36 (0.22, 0.49)	<0.001	418 (42.9)	1740 (53.3)	0.21 (0.14, 0.28)	<0.001
Diabetes	1289 (24.8)	74 (23.0)	167 (26.1)	0.07 (-0.06, 0.21)	0.299	230 (23.6)	818 (25.1)	0.03 (-0.04, 0.11)	0.345
Dyslipidemia	3733 (71.8)	241 (74.8)	460 (71.8)	0.07 (-0.06, 0.20)	0.311	689 (70.7)	2343 (71.8)	0.03 (-0.05, 0.10)	0.481
CVD	292 (5.6)	9 (2.8)	38 (5.9)	0.15 (0.02, 0.29)	0.033	40 (4.1)	205 (6.3)	0.10 (0.03, 0.17)	0.010
Medication, N (%)									
Antihypertensive drugs	1689 (32.7)	81 (25.2)	243 (38.0)	0.28 (0.14, 0.41)	<0.001	265 (27.3)	1100 (34.0)	0.15 (0.07, 0.22)	<0.001
Hypoglycemic drugs	572 (11.0)	35 (10.9)	72 (11.2)	0.01 (-0.12, 0.15)	0.859	106 (10.9)	359 (11.0)	0.00 (-0.07, 0.08)	0.907
Lipid-lowering drugs	555 (10.8)	43 (13.5)	74 (11.6)	0.06 (-0.08, 0.19)	0.402	98 (10.2)	340 (10.5)	0.01 (-0.06, 0.08)	0.760
Death endpoint, N (%)									
All-cause mortality	320 (6.2)	2 (0.6)	62 (9.7)	0.42 (0.28, 0.55)	<0.001	22 (2.3)	234 (7.2)	0.23 (0.16, 0.31)	<0.001
CV mortality	107 (2.1)	1 (0.3)	17 (2.7)	0.19 (0.06, 0.33)	0.011	5 (0.5)	84 (2.6)	0.17 (0.10, 0.24)	<0.001

BMI: body mass index; CV: cardiovascular; CVD: cardiovascular disease; eGFR: estimated glomerular filtration rate; Hcy: homocysteine; IQR: interquartile range; MTHFR: methylenetetrahydrofolate reductase; SD: standard deviation; SMD: standardized mean difference.
